# Effect of the Lipid Landscape on the Efficacy of Cell-Penetrating Peptides

**DOI:** 10.3390/cells12131700

**Published:** 2023-06-23

**Authors:** Florina Zakany, István M. Mándity, Zoltan Varga, Gyorgy Panyi, Peter Nagy, Tamas Kovacs

**Affiliations:** 1 Department of Biophysics and Cell Biology, Faculty of Medicine, University of Debrecen, 4032 Debrecen, Hungary; florina.zakany@med.unideb.hu (F.Z.); veze@med.unideb.hu (Z.V.); panyi@med.unideb.hu (G.P.); 2 Department of Organic Chemistry, Faculty of Pharmacy, Semmelweis University, 1085 Budapest, Hungary; mandity.istvan@ttk.hu; 3 TTK Lendület Artificial Transporter Research Group, Institute of Materials and Environmental Chemistry, Research Centre for Natural Sciences, 1117 Budapest, Hungary

**Keywords:** cell-penetrating peptides, lipid rafts, membrane biophysics, membrane fluidity, cholesterol, diabetes mellitus, Alzheimer’s disease

## Abstract

Every cell biological textbook teaches us that the main role of the plasma membrane is to separate cells from their neighborhood to allow for a controlled composition of the intracellular space. The mostly hydrophobic nature of the cell membrane presents an impenetrable barrier for most hydrophilic molecules larger than 1 kDa. On the other hand, cell-penetrating peptides (CPPs) are capable of traversing this barrier without compromising membrane integrity, and they can do so on their own or coupled to cargos. Coupling biologically and medically relevant cargos to CPPs holds great promise of delivering membrane-impermeable drugs into cells. If the cargo is able to interact with certain cell types, uptake of the CPP–drug complex can be tailored to be cell-type-specific. Besides outlining the major membrane penetration pathways of CPPs, this review is aimed at deciphering how properties of the membrane influence the uptake mechanisms of CPPs. By summarizing an extensive body of experimental evidence, we argue that a more ordered, less flexible membrane structure, often present in the very diseases planned to be treated with CPPs, decreases their cellular uptake. These correlations are not only relevant for understanding the cellular biology of CPPs, but also for rationally improving their value in translational or clinical applications.

## 1. Introduction

While the cell membrane is relatively freely penetrable for small, hydrophobic molecules, it presents an impenetrable barrier for most other kinds of compounds. Using energy-independent direct translocation or biological processes, membrane-active peptides are able to traverse this obstacle. Membrane-active peptides are divided into two main categories: antimicrobial peptides (AMPs) and cell-penetrating peptides (CPPs). AMPs (also known as host defense peptides or HDPs) are part of the innate immune response as potent, broad-spectrum antibiotics acting through the destabilization of membranes of the pathogens. Unlike AMPs, CPPs can translocate into living cells and their organelles without lasting damage (unless applied in extremely high concentrations) [1]. This peculiarity makes CPPs suitable to deliver drugs and other compounds to intracellular targets or across the blood–brain barrier [2].

CPPs can be categorized based on several criteria, including origin, backbone and composition just to mention a few. The first CPPs had a natural origin, isolated from living organisms. The basic region of the HIV Tat protein, encompassing amino acids 38–58, was shown to be responsible for the membrane translocation capability of the full-length protein [3]. Similarly, the antennapedia motif of *Drosophila* homeoproteins is also capable of crossing membranes, and this property has been mapped to the third helix of the protein [4,5]. This 16-amino acid segment was later named penetratin. By modifying the sequence of natural CPPs, semi-synthetic CPPs have also been produced. While the cell penetrating potential of CPPs was originally assumed to be a peculiar property, a large number of peptide sequences, with confirmed and putative membrane penetration capability have been identified in human proteins as well [6]. Furthermore, novel, purely synthetic, or partially natural chimeric peptides were also constructed. As far as their structure is concerned, most CPPs have a linear backbone, but cyclic peptides can also be found among both the natural and the synthetic ones. CPPs do not have a well-defined consensus sequence, and their membrane penetration capability cannot be linked to any specific amino acid either. However, some peculiar features can be identified in their sequence: most of them contain multiple cationic amino acids, but many sequences have mixed types of residues with amphipathic or hydrophobic chains [7]. This observation led to the most widespread classification of CPPs based on their charge and hydrophobicity. According to this taxonomy, most CPPs are classified as cationic, while the second largest group comprises amphipathic peptides in which hydrophobic and hydrophilic amino acids are typically not randomly distributed, but they are rather sequestered either in the primary or secondary structure. Two relatively small groups of CPPs exhibit either anionic or purely hydrophobic character. However, it must be noted that there is an overlap between these four different classes, i.e., certain CPPs belong to more than one group [8,9].

**Figure 1:**
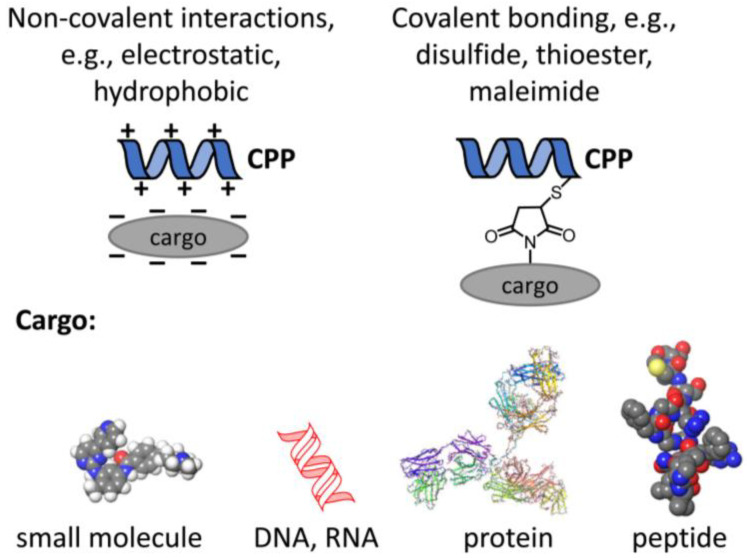
Principles of coupling cargo to CPPs. In order to fulfill their medical potential, CPPs have to be conjugated to therapeutically relevant cargo like small molecules, e.g., kinase inhibitors, nucleic acids, or proteins as large as antibodies or antibody fragments or peptides. The conjugation mechanism may be based on non-covalent or covalent interactions.

The interest in CPPs stems from the fact that they can transport large and hydrophilic cargos ranging from small molecules to large proteins to cells (Figure 1). The overarching aim of this review is to discuss the effect of CPPs in the context of how they interact with the membrane. To this end, some general biophysical properties of the plasma membrane will be presented followed by a brief introduction to the uptake mechanisms of CPPs. These introductory paragraphs will allow us to present our view how fundamental biophysical properties of membranes determine and modify the uptake of CPPs, and how the very same principles limit their potential therapeutic value in certain diseases in which they are seen as magic bullets.

## 2. Biophysical Parameters and Lipid Rafts of Cellular Membranes

The fact that cellular entry mechanisms of CPPs involve permeation through cellular membranes provides ample opportunities for modulatory permissive and cooperative membrane–CPP interactions. This section of the review is devoted to discussing the general biophysical properties of membranes relevant from the standpoint of how CPPs might insert into and penetrate membranes.

In general, the modulatory actions of lipids on proteins and peptides, including CPPs, can be mediated by direct binding, alterations in membrane biophysical parameters such as membrane fluidity, hydration, thickness, elasticity and dipole potential, or, most commonly, by a spectrum of these effects [10,11,12]. Although the aforementioned membrane biophysical parameters are intrinsically related to each other, they characterize different aspects of molecular organization in bilayers. Membrane fluidity and hydration are two strongly connected, order-related parameters of bilayers. In a narrower sense, membrane fluidity is defined as the degree of diffusion freedom of membrane-embedded molecules, while membrane hydration is the extent of water penetration into the hydrophobic regions of bilayers. Therefore, both parameters characterize lipid packing although at different depths of the membrane, i.e., fluidity in the central region while hydration closer to the membrane–water interface, and consequently, are often referred to collectively as “lipid packing” or “membrane order”. The fundamental determinant of this bilayer property is the type of phospholipids because straight, saturated acyl chains enable their close packing, and thus, increasing order, while the presence of cis bonds in unsaturated fatty acids makes the chains bend, creating extra space between hydrocarbon chains leading to larger area per molecule and decreased packing [13,14,15]. Consistently, incorporation of polyunsaturated fatty acids was shown to significantly decrease membrane order [16,17,18]. On the contrary, the level of membrane cholesterol shows strong positive correlation with lipid packing due to its ordering effects on acyl chains [18,19,20,21]. Increases in the amounts of glucosylceramides and ceramides were similarly demonstrated to reduce membrane fluidity and hydration, especially when containing long and saturated acyl chains [22,23,24]. Consistent with their effects on hydrocarbon chains of phospholipids, bilayer-ordering lipids increase, while fluidizing lipids decrease hydrophobic thickness of biological membranes [17,25,26,27]. This parameter is essential in the modulation of membrane–peptide or –protein interactions according to the hydrophobic mismatch theory. If proteins or peptides get embedded into a membrane and their hydrophobic thickness does not match that of lipid constituents, energetically unfavorable mismatch arises, which induces adaptation mechanisms. These involve changes in lipids such as straightening their acyl chains or preferential aggregation, and peptides or proteins including their oligomerization, aggregation, tilting, and alterations in secondary structure and conformation [10,11,28]. These changes can influence the efficiency of the interactions of peptides with bilayers and their transmembrane permeation as discussed later.

Spontaneous curvature is another property of bilayers related to their composition. The major determinant of whether a lipid molecule induces curvature is the width of the hydrophilic head relative to the hydrophobic part. Cylinder-shaped lipids having an approximately matching width of the hydrophilic and hydrophobic parts, e.g., phosphatidylcholine (PC), favor flat bilayer geometry. Lipids with a wider hydrophobic part, e.g., phosphatidylethanolamines (PE) or ceramides, are termed cone-shaped, while inverted cone-shaped lipids, such as lysophospholipids or gangliosides, have wider hydrophilic heads. Both lipid classes curve the membrane, cone-shaped and inverted cone-shaped molecules toward the hydrophilic and hydrophobic layers, respectively [29,30,31,32]. The tendency for spontaneous curvature is substantially modulated by the presence of polyunsaturated fatty acids that increase elasticity [33], and cholesterol that, on the contrary, increases the interfacial elastic area/compression moduli and bending stiffness [19,25,34], modifies the intrinsic spontaneous curvature of membranes [35,36] and preferentially accumulates in high-curvature regions [37,38]. However, if such curvature cannot be fulfilled for example because of the presence of hydrophobic mismatch with proteins, curvature elastic stress (frustration) arises, which can affect peptides and proteins through altering the stability of certain conformations or secondary structures. In turn, peptides and proteins can modify intrinsic curvature stress of lipids favoring or disfavoring spontaneous curvature of bilayers [10,28,39]. Given that curved regions are the primary targets of membrane fusion and fission, intrinsic spontaneous curvature of phospholipids and its modification by the presence of transmembrane proteins and other lipids such as cholesterol, glucosylceramides, and ceramides can largely affect membrane insertion and cellular uptake of CPPs as described later.

Membrane dipole potential (DP) is probably the most enigmatic biophysical property of biological bilayers, which arises due to the non-random orientation of molecular dipoles of carbonyl groups of phospholipids and cholesterol and water molecules at the interfacial region. This alignment results in the generation of a large potential, positive in the hydrophobic region, and a consequent immense intramembrane electric field [10,40,41]. DP is determined by the membrane lipid composition such as the amounts of different phospholipids since both the chemical type of their headgroup and the saturation of their acyl chains influence its magnitude [18,42]. Membrane cholesterol is also a fundamental determinant as its level shows strong unequivocal positive correlation with the value of DP [18,43,44,45,46]. Furthermore, increases in the glucosylceramide levels also elevate DP [47]. Due to DP-associated enormous electric field and the generally non-uniform charge distribution in proteins, the DP can modulate the function of transmembrane proteins by affecting their conformational stabilities [10,40,41,48,49]. In accordance, the DP can influence the membrane binding and translocation of charged CPPs as can be seen in the following sections of the review.

An additional level of molecular organization in bilayers is provided by its lateral heterogeneity, i.e., the presence of membrane nanodomains characterized by unique lipid composition and membrane biophysical properties. Lipid rafts are cholesterol- and (glyco)sphingolipid-enriched nanodomains in the cell membrane that are stabilized by lipid–lipid and lipid–protein interactions and preferentially accumulate certain proteins to facilitate their interactions while segregating others [10,50,51,52]. While the initial lipid raft hypothesis emphasized the role of lipid-mediated organization, the actin cytoskeleton can also actively organize the lateral heterogeneity of membranes according to the picket-fence model [53,54]. Their investigation in live cells without perturbing their physiological state revealed that they are short-lived (couple of ms) and their size is exceedingly small (<50 nm), although they can be stabilized by various factors, e.g., during transmembrane signaling [55]. Hence, they are referred to as nanodomains in the current manuscript. Due to their special composition, lipid rafts are characterized by a distinctive membrane biophysical microenvironment with lower fluidity and hydration, increased thickness and elevated DP, which can influence the structure and function of proteins and peptides as described above [22,46,47,56]. These specialized regions are involved in a great variety of cellular processes such as signal transduction, cell adhesion and migration, synaptic transmission, pathogen entry, amyloid formation, and endocytic mechanisms [10,50,52]. The involvement of lipid rafts in endocytosis provides a foundation for their relevance for CPP uptake. Consistently, their altered level, composition and structure are often observed in diseases such as cancer, metabolic, or neurodegenerative disorders [52,57,58], which can influence the applicability of CPPs in these conditions.

## 3. Models for Direct CPP Permeation

While an in-depth discussion of CPP uptake mechanisms is not the primary topic of the current review, putting the effects of membrane biophysical parameters into context requires a brief overview of these processes. CPPs can enter cells through energy-independent direct translocation and energy-dependent endocytic mechanisms. Although some publications attributed apparent direct CPP permeation to fixation-induced artifacts [59,60], subsequent reports convincingly demonstrated passive direct translocation of CPPs with their diffuse intracellular accumulation in the absence of membrane disruption observed especially at higher peptide concentrations even when endocytosis was blocked [61,62,63,64,65,66]. At the same time, endocytic uptake was demonstrated to dominate at low peptide concentrations in most cases with the exception of penetratin [61,63,64,67,68,69,70]. These findings suggested that both endocytic uptake and direct permeation of CPPs can occur depending on the given conditions such as cell type, chemical modifications and concentrations of CPPs, presence and types of cargo, incubation time, and temperature, etc., as reviewed recently [71,72,73,74].

**Figure 2:**
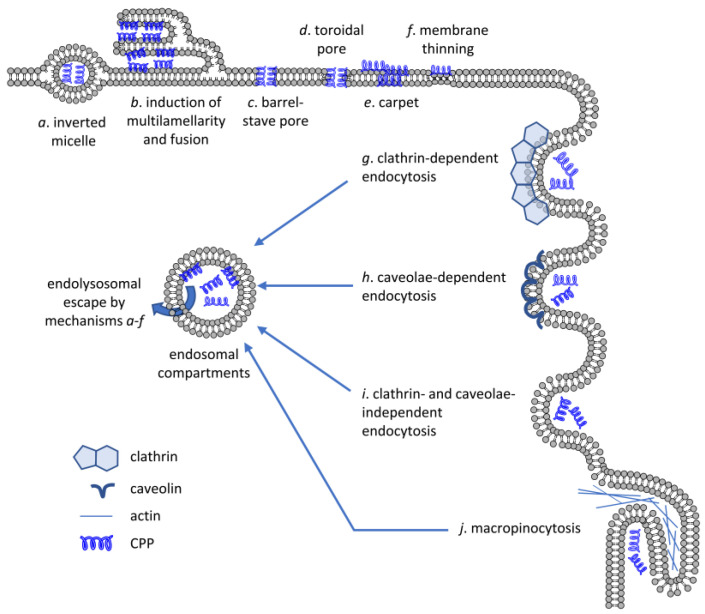
Mechanisms of CPP uptake. CPPs are capable of crossing artificial lipid bilayers or the membrane of living cells by energy-independent mechanisms (**a**–**f**) or by energy-dependent means requiring active participation of the cell (**g**–**j**). Several different mechanisms have been put forward to explain direct membrane crossing of CPPs including inverted micelle formation, induction of multilamellarity, barrel-stave or toroidal pore formation, carpet formation and membrane thinning. Biologically active, energy-dependent uptake mechanisms include endocytosis pathways dependent on clathrin or caveolae, clathrin- and caveolae-independent endocytosis and micropinocytosis. Even if CPPs enter cells by one of the energy-dependent mechanisms, they must cross the membrane of the endolysosomal compartment by one of the direct translocation mechanisms to reach the cytosolic compartment.

When examining direct translocation, the initial phase of cytoplasmic entry occurred at small spatially restricted transient regions where CPP bound to negatively charged cell surface proteoglycans and phospholipids suggesting local membrane inversion, and thus, alterations in membrane structure [61,62,75]. Five mechanisms were proposed to mediate direct CPP penetration as reviewed in detail elsewhere [73,74,76,77]: (i) inverted micelle formation; (ii) the carpet-like model; (iii) the membrane thinning model; (iv) the transient pore formation; and (v) induction of multilamellarity and the stalk–pore hypothesis (Figure 2). Their common feature is that all of them involve transient membrane perturbation.

(i) Inverted micelle formation was suggested to involve interactions between Trp residues and the hydrophobic core of the membrane resulting in the encapsulation of the peptides in the hydrophilic cavity. However, this mechanism was later deemed unlikely for highly cationic CPPs.
(ii) According to the carpeting model, negatively charged headgroups of bilayer phospholipids compensate for the electrostatic repulsion between positive charges of CPPs resulting in the peptide monomers lying parallel to the surface of the membrane and self-associating with other monomers in a carpet-like fashion with the hydrophobic regions of the peptide embedded into the bilayer. This structure can in turn perturb the structural organization of the membrane leading to transmembrane leakage and detergent-like disintegration.
(iii) In the membrane thinning model, similar electrostatic interactions between negatively charged phospholipids and cationic CPPs, and bilayer stretching due to peptide–peptide interactions collectively result in thinning of the membrane. While inverted micelle formation, carpeting and barrel–stave pore formation seems to mediate direct membrane translocation of AMPs and amphipathic CPPs, thinning and toroidal pore formation are more commonly suggested in the literature for highly cationic and less amphipathic CPPs. However, discrimination between these strongly related mechanisms seems arbitrary, and most probably, their combination occurs, and different mechanisms may even represent various steps of the same process. In accordance with this hypothesis, Tat—due to the interaction between the positively charged guanidinium components of the arginines and the phosphate groups of phospholipids—induced membrane thinning and bending elasticity in lipid bilayers [78]. The presence and type of cargo attached to CPPs can modulate their effects on lipid packing and membrane thickness. For example, different degree of bilayer insertion and consequent thinning was observed when studying penetratins conjugated to fluorophores of various size and hydrophobicity [79]. CPPs can also reorganize lateral distribution of lipids as demonstrated by the preferential recruitment of phospholipids with unsaturated and shorter acyl chains by penetratin while segregation of those with saturated and long hydrocarbons [80].
(iv) Transient pore formation can happen according to the barrel–stave and toroidal pore models. In the former, CPPs form a barrel with their hydrophobic residues interacting with lipid chains and the hydrophilic residues lining the pore. In the latter, due to the bending of lipids driven by electrostatic interactions between phosphates and positive peptide residues, CPPs are always close to lipid headgroups and both CPPs and lipids line the pore itself. In both cases, pores appear only above a critical CPP concentration, often following CPP-induced membrane thinning resulting in large distortions of bilayer organization by attracting more headgroups via multidentate hydrogen bonds from the same leaflet and, due to thinning, even from the opposite layer. Furthermore, when a critical concentration is reached, an Arg residue translocates to the distal layer and nucleates the formation of a water pore that closes after a short period of time (within microseconds) preventing damage and nonspecific leakage. The peptides can diffuse on this pore surface to get to the inner leaflet and eventually to the aqueous solution [81]. Such toroidal pores were indeed demonstrated in crystallographic studies of Tat [82] and oligoarginine peptides, and their presence was confirmed in model and cellular membranes [83]. For transient pores to form, the membrane must exhibit negative Gaussian curvature, which is the result of an intricate balance in amino acid composition of CPPs with lysines creating negative curvature, hydrophobic amino acids inducing positive curvature, and arginines simultaneously inducing positive and negative curvature along the two perpendicular principal directions [84]. Based on molecular dynamics (MD) simulations, various studies also supported that pore formation can be facilitated by the transmembrane potential gradient by reducing the energy cost of crossing the bilayer [85,86]. MD simulations and live cell experiments demonstrated that membrane binding of cationic Tat peptides carrying a cargo can lead to megapolarization decreasing the transmembrane potential to as low as –150 mV, which largely lowers the free energy barrier associated with CPP translocation [87].
(v) While remaining widely accepted, toroidal pore formation has been questioned recently by several studies proposing that cationic CPPs passively enter vesicles and live cells by inducing membrane multilamellarity and fusion. CPP entry at spatially restricted regions in the plasma membrane proceeds by the formation of “endocytosis-like” lipidic tubular structures [75,88,89]. These findings led to the hypothesis that passive CPP permeation can happen according to a membrane fusion-based mechanism involving a stalk intermediate, followed by formation of a hemifused structure and opening of a fusion pore, similar to that described for the fusion of vesicles with the plasma membrane in calcium-triggered exocytosis, which was supported by cryo-electron microscopy and interbilayer Förster resonance energy transfer (FRET) [90].

## 4. The Effects of Membrane Biophysical Parameters on Direct CPP Permeation

### 4.1. Effects of Lipid Packing on Direct CPP Entry

**Figure 3:**
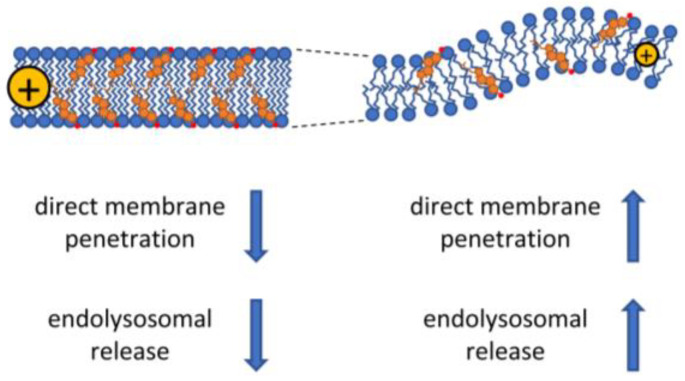
The effect of the biophysical properties of membranes on CPP uptake. Cholesterol and saturated fatty acids cause decreased membrane fluidity also associated with increased membrane thickness and higher dipole potential. These biophysical changes generally lead to less efficient cellular uptake of CPPs by both the energy-dependent and -independent pathways, although caveolae-dependent endocytic uptake of CPPs may be enhanced under these conditions due to the cholesterol-dependence of caveolae. On the contrary, low concentration of cholesterol in the membrane and the presence of unsaturated fatty acids lead to more fluid membrane structure associated with smaller membrane thickness, a higher propensity for curvature, and lower dipole potential. These alterations are thought to enhance both the direct and endocytosis-dependent CPP uptake mechanisms.

Given that most direct permeation mechanisms involve structural rearrangements and bending of membrane phospholipids, these processes are intrinsically connected to biophysical parameters such as lipid packing, spontaneous curvature, and bending rigidity of the cell membrane. It is reasonable to assume that higher lipid bilayer thickness, packing order, and bending stiffness are expected to act against entry that can occur only if the membrane is thin and flexible enough (Figure 3). Pore formation in general is much less favorable in thicker bilayers as demonstrated by MD simulations of PC bilayers formed by lipids of varying acyl chain length [91]. Accordingly, Tat was demonstrated to preferentially insert into loosely packed regions in monolayer expansion measurements [92] and penetratin induced curvature in liquid-disordered PC but not raft-like ordered sphingomyelin/cholesterol multilamellar vesicles [93]. Confocal microscopy and flow cytometry analysis of a variety of fluorophore-conjugated CPPs in giant unilamellar vesicles (GUVs), devoid of cellular energy-dependent mechanisms and actin cytoskeleton, showed that all CPPs preferentially associated with liquid-disordered membrane areas and accumulated in the lumen even at low temperatures without the nonspecific uptake of hydrophilic tracers. Furthermore, methyl-β-cyclodextrin (MβCD)-induced cholesterol extraction enhanced, while cholesterol loading using cholesterol-MβCD complexes reduced CPP entry [61,94,95]. This observation is in keeping with studies demonstrating that lower cholesterol content or filipin-mediated cholesterol sequestration of giant plasma membrane vesicles elevated CPP uptake [96]. Penetratin also demonstrated strong preference for phospholipids of disordered domains with unsaturated or short saturated acyl chains in multilamellar vesicles of varying phospholipid content [97].

### 4.2. Effects of Spontaneous Curvature and Bending Elasticity on Direct CPP Entry

In crystallographic studies of aqueous solutions of simple phospholipids and Tat peptides, supporting the role of the spontaneous curvature of bilayers in the translocation, CPPs entered only unilamellar vesicles containing DOPE with a negative intrinsic curvature and not those only formed by phospholipids with zero intrinsic curvature (dioleoyl-phosphatidylcholine (DOPC), dioleoyl-phosphatidylserine (DOPS), or dioleoyl-phosphatidylglycerol (DOPG)) [82]. Similarly, when examining interaction of fluorescently labeled Tat with PC/cholesterol GUVs, rapid translocation and pore formation was observed only in the presence of PE [98]. In MD simulations of dipalmitoyl-phosphatidylcholine/dipalmitoyl-phosphatidylserine (DPPC/DPPS) bilayers, cholesterol hindered toroidal pore formation and Tat permeation by stabilizing liquid-ordered phases, increasing membrane stiffness and weakening favorable peptide–lipid interactions [99].

While negative curvature is mostly accepted to contribute to CPP entry, treatment of GUVs and living cells with the N-terminal 18-residue peptide of epsin-1 (EpN18), which induces positive curvature in bilayers, also promoted the direct penetration of a fluorescently labeled octa-arginine, even when conjugated with the proapoptotic domain peptide PAD, as observed by diffuse staining in confocal microscopy [100]. Pyrenebutyrate, a hydrophobic ion that was originally thought to act as an ion-pairing charge scavenger through hydrophobic complex formation with arginine-rich CPPs, exerted similar effects on direct entry of octa-arginine and Tat into GUVs by increasing fluidity and curvature [101]. While these studies emphasized the role of positive curvature induction for EpN18 and pyrenebutyrate, a recent report proposed an alternative mechanism of action. While the tendency of these treatments to facilitate curvature and elevate octa-arginine uptake was confirmed in living cells, these effects were accompanied by significant reductions in lipid packing, especially at foci of CPP entry, and the penetration of octa-arginine was associated with further loosening. Based on these findings it was proposed that loosening of lipid packing can be a common and fundamental mechanism facilitating the uptake of arginine-enriched CPPs by strengthening the hydrophobic interactions of the peptide backbone with lipid acyl chains [102]. Application of trimeric form or conjugation with a pyrenebutyryl moiety even enhanced EpN18 effects on membrane properties and octa-arginine uptake [103,104]. Furthermore, membrane curvature modulation of GUVs and erythrocytes by osmotic pressure was recently demonstrated to affect octa-arginine entry with hypotonic and hypertonic conditions promoting and suppressing, respectively, curvature and CPP entry. It was suggested that local and temporal positive curvature changes may also promote CPP entry [105].

## 5. Active, Energy-Dependent Uptake of CPPs

### 5.1. Evidence for the Involvement of Endocytosis and Proteoglycans in CPP Uptake

There is widespread agreement in the literature that CPPs enter cells by active, energy-dependent endocytic processes at low concentrations [73]. This section will outline the experimental evidence supporting these claims followed by arguments presented in Section 6 that the biophysical properties of membranes also impact this kind of cellular uptake.

The importance of endocytic processes in the cellular uptake of CPPs stem from the fact that the overwhelming majority of CPPs are endocytosed in living cells at low to moderate concentrations [61,63,64,67,68,69,70]. CPPs typically bind membranes, and direct membrane penetration processes are obviously initiated in live cells as well, but the speed of membrane turnover is so fast that endocytosis usually precedes direct translocation processes. Several different endocytic processes have been implicated in the cellular uptake of CPPs (Figure 2), among which macropinocytosis has been observed the most commonly. The process of macropinocytosis does not begin with conventional membrane invagination, but is rather the consequence of enhanced membrane ruffling often associated with cellular activation [106]. These membrane protrusions do not envelop a ligand-coated particle, but instead collapse onto and fuse with the plasma membrane to generate the macropinosomes [107]. A large body of experimental evidence implies the involvement of macropinocytosis in the uptake of several CPPs [108,109].

Besides macropinocytosis, other endocytic processes also contribute to the energy-dependent uptake of CPPs. Cellular entry of Tat and transportan has been shown to depend on caveolar endocytosis according to colocalization and gene knock-down experiments [67,110]. Although some studies explicitly rejected the possible role of clathrin-mediated endocytosis in CPP uptake, it has been repeatedly implicated by others. Partial inhibition of the uptake of Tat was achieved by blockers of clathrin-dependent endocytosis implying the nonexclusive role of this endocytic pathway in the uptake process [111]. A comprehensive study concluded that penetratin, Tat and nona-arginine use all three major endocytic processes, clathrin- and caveolae-dependent endocytosis and macropinocytosis, but the extent to which they rely on each of these processes differs [61]. While a thorough investigation of several CPPs confirmed the previous conclusion, the authors even noted that the endocytic process used also depends on the peptide concentration [112].

There is consensus that initiation of CPP uptake by any of the aforementioned endocytic processes is preceded by binding of the peptide to the plasma membrane. While several membrane receptors have been implicated in the process, a general picture seems to emerge according to which cell surface proteoglycans carrying negatively charged glycosaminoglycans bind the positively charged residues of CPPs [68,111,113]. Electrostatic binding of CPPs to glycosaminoglycans is not simply a passive event in the uptake process, but it is assumed to induce endocytosis by bringing about membrane protein clustering. Heparan sulfate bearing membrane proteins have been suggested to function as general endocytosis receptors, and the type of endocytosis they induce may depend on the protein they are present in [114]. Accordingly, syndecan was suggested to be involved in clathrin-mediated endocytosis or macropinocytosis, while another heparan sulfate-containing protein, glypican, was implicated in inducing caveolae-dependent uptake. Besides cell surface glycosaminoglycans taking the center stage as the membrane receptors of CPPs, other proteins, including Neuropilin-1, chemokine, and scavenger receptors have all been implicated to contribute to the process [115,116,117].

### 5.2. Endolysosomal Escape of CPPs

Even if different endocytic mechanisms have been implicated in the uptake of CPPs, many of these routes converge at the early/sorting endosomal stage [118]. Furthermore, endocytosed CPPs must cross the membrane of the endosomal compartment to reach the cytosolic-nuclear compartment to exert their action. This step, termed endolysosomal escape, is the limiting factor determining the efficiency of CPPs. Compared to the direct and endocytic uptake mechanisms, relatively little is known about endolysosomal escape. One of the reasons for the scarcity of information on this topic is the difficulty to measure it. Punctate and diffuse intensity distribution of fluorescence correspond to endocytic and cytoplasmic accumulation of fluorescently labeled CPPs, respectively, but it is a challenging image analysis task to quantitate them [119]. Furthermore, both cargos and the fluorescence label modify the distribution of CPPs in vivo and in vitro, further complicating the interpretation of results [120,121]. Mass spectrometry, potentially combined with cell fractionation, can be used to accurately determine the concentration of CPPs in different compartments. Furthermore, degradation of CPPs can also be readily spotted by mass spectrometry, which is a great asset given that the failure to escape from the endolysosomal compartment leads to lysosomal degradation [122,123]. The pH-sensitive fluorescence of certain fluorophores can also be used for assessing how large fraction of CPPs is present in the cytosol. Naphthofluorescein-labeled CPPs, whose fluorescence is quenched at the acidic pH of the endosomal compartment, combined with the application of CPPs conjugated to pH-insensitive fluorophores can be used for quantitating the cytosolic and endosomal concentrations of CPPs [18,43,124]. Endosomal escape was also measured by applying the principle of split luciferase assay. In this method, half of luciferase is localized to the cytosol because it is fused to actin. The other half of luciferase is conjugated to the CPP, and if it gets to the cytosol after endolysosomal escape, the two molecular halves complement each other producing bioluminescence [125].

Relatively little attention has been paid to the molecular details of endolysosomal escape. Although there are differences between the lipid composition of the plasma membrane and endosomes, they share many lipid species and the change in lipid composition during the migration and maturation of endosomes must be gradual [126]. Therefore, it is a reasonable assumption that the membrane crossing mechanisms outlined in Section 3 (Figure 2a–f) also apply to endolysosomal escape. It also seems obvious that membrane penetration and endocytosis of CPPs are competitive processes, and whether a CPP crosses the plasma membrane or the membrane of endosomes also depends on the relative speed of membrane penetration and endocytosis. A recent study concluded that the majority of membrane crossing of CPPs is attributable to direct translocation across the plasma membrane [127], although this finding is at odds with the multitude of results suggesting that inhibition of endocytosis substantially inhibits cellular uptake of CPPs, including their accumulation in the cytosol [43].

**Figure 4:**
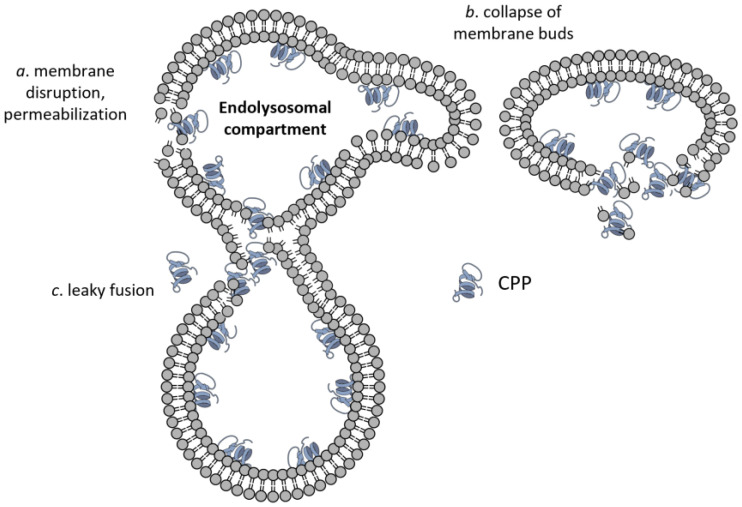
Endolysosomal escape of CPPs. Besides the membrane translocations mechanisms shown in Figure 2, three modes of CPP escape from the endolysomal compartment have been suggested, which are membrane permeabilization (**a**), formation and collapse of membrane buds (**b**), and leaky fusion (**c**).

Besides the routes for plasma membrane crossing, several specific molecular mechanisms have been proposed for endosomal escape. All of them involve some sort of phase transition/membrane destabilization leading to membrane permeabilization [128], formation and rupture of membrane buds from endosomes [129], or the leaky fusion of endosomes [130] (Figure 4).

Besides the aforementioned mechanisms based on the inherent membrane penetration potential of CPPs, several tricks have been applied to increase endolysosomal escape. The positive intramembrane DP, described in Section 2, has been shown to limit the membrane translocation of positively charged CPPs in model membranes and in live cells as a result of electrostatic interactions [43,131]. Building upon this correlation, treatments decreasing the DP are expected to enhance membrane crossing of CPPs either at the plasma membrane or in the endolysosomal compartment. Consistently, the applications of statins, inhibiting cholesterol biosynthesis—and thereby lowering membrane cholesterol content—and ω-3 fatty acids increased the cellular uptake of penetratin in cancer cell lines [18,43]. Both treatments decreased DP by partially subverting the orderly arrangement of lipids. While the previous approach achieves enhanced endosomal escape and membrane penetration by modifying membrane properties, the CPPs themselves have been modified in several ways to reach this aim. The purpose of these modifications is two-fold. They are aimed at: (i) enhancing endosomal escape and (ii) releasing the cargo from the CPP after the successful escape. Tat fused to two membrane-active peptides, cecropin and melittin, or to the fusogenic influenza virus haemagglutinin, was able to deliver cargo to cells efficiently by lysing endosomes without toxicity [70,132,133]. The incorporation of fusogenic dioleoyl-phosphatidylethanolamine into liposomes decorated with octa-arginine significantly increased the transfection efficiency of DNA enclosed into the liposome. Further molecules, e.g., for targeting or for enhanced half-life in circulation, can be incorporated into the liposomal membrane turning the particles into multifunctional envelope-type nano devices (MEND) [134]. Furthermore, another approach to enhance the endosomal escape activity of CPPs is to induce their multimerization, which results in higher endosomolytic potential. Such oligomerization has been reported by the application of multimerization domains and dendrimers [135,136]. Dimerization of Tat induced by a disulfide bond not only enhances the endosomal escape of the CPP, but reduction of the disulfide bridge in the cytosol also releases monomeric Tat [137]. The principle of breaking of disulfide bonds in the reductive environment of the cytosol can also be used for releasing CPP-bound therapeutic cargo if the drug is coupled to the CPP by a disulfide bond. Despite the great promise of and the immense amount of work invested into CPPs, their potential has so far been translated only to a limited success with low endosomal escape and lack of specificity being the most important factors to blame [138]. Future developments along the lines outlined in this section may improve the in vivo applicability of CPPs.

## 6. Membrane Effects on Endocytic Uptake of CPPs

### 6.1. The Effect of Membrane Biophysical Properties on Endocytic Uptake of CPPs

While the exact molecular mechanisms of the endocytic uptake and escape mechanisms of CPPs are different, all of them involve bilayer bending and invagination, and thus, they are also intrinsically connected to the spontaneous curvature and bending rigidity of the cell membrane. Consequently, alterations in these biophysical parameters, as discussed in Section 4 (Figure 3), are expected to strongly affect the endocytic uptake of CPPs. The common tendency of CPPs to induce negative Gaussian curvature can help the process of endocytosis as well. To support this hypothesis, MD simulations of DPPC and DOPC bilayers showed that binding of multiple penetratin or Tat peptides can stimulate pinocytosis by inducing large-scale curvature of the membrane persisting throughout the timescale of the simulations (hundreds of nanoseconds), and ultimately, leading to formation of small vesicles encapsulating the peptides without the appearance of pores [139]. Tat was reported to induce negative Gaussian curvature and cytoskeletal reorganization reminiscent of those in macropinocytosis in an actin-dependent manner in actin-encapsulated giant vesicles without receptors [84]. Surprisingly, disruption of actin organization using cytochalasin D, Latrunculin B, or Jasplakinolide was demonstrated by flow cytometry to exert opposing effects on the entry of fluorophore-conjugated octa-arginines, as it dramatically increased the uptake of EGFP-octa-arginine and the fluid-phase probe dextran while strongly inhibited that of octa-arginine conjugated to Alexa Fluor 488 in A431 skin epithelial cells. On the other hand, actin breakdown with cytochalasin D reduced the uptake of oligoarginines with both conjugates in HeLa cells. Diffuse staining pattern was not observed in any of the two cell types arguing against direct permeation. These observations demonstrate that even the same CPP can use different routes of endocytosis in different cells, or even in the same cell type depending on the presence and type of cargo, and the actin cytoskeleton can act as a gateway or a barrier even for strongly related CPP-cargo constructs [140].

### 6.2. Dual Role of Lateral Membrane Organization in the Endocytic Uptake of CPPs

As described in previous sections, lipid rafts, the most widely known nanodomains in lateral membrane organization, are expected to play dual roles in the cellular uptake of CPPs. On the one hand, these nanodomains are involved in caveolae- and lipid raft-mediated endocytosis and lipid raft-mediated macropinocytosis. Therefore, rafts can act as primary sites for CPP entry. Consistently, their disruption by MβCD or nystatin was shown to reduce CPP uptake under certain circumstances [61,67,70,95,108].

On the other hand, their unique membrane biophysical microenvironment characterized by higher order of lipid packing and elevated DP can interfere with direct CPP permeation and also endolysosomal escape, and lipid raft disruption might in fact facilitate these processes [61,94,95]. Therefore, the result of these counteracting effects mediated by these nanodomains strongly depend on the conditions of the CPP application (types of target cells and their cell surface receptors, type of CPPs and attached cargos, peptide concentrations, incubation time, temperature, pH, etc.) and elucidating the exact molecular mechanisms of CPP entry is crucial to predict the effects induced by alterations in the lateral heterogeneity of membranes. As an example for illustrating the dual role of lipid rafts and cholesterol, treating living cells with cholesterol-depleting MβCD stimulated their direct permeation while inhibiting Tat and oligoarginine uptake via endocytic mechanisms, [61,95].

## 7. Potential Therapeutic Applications of CPPs and Their Limitation by Membrane Biophysical Alterations in Diseases

**Figure 5:**
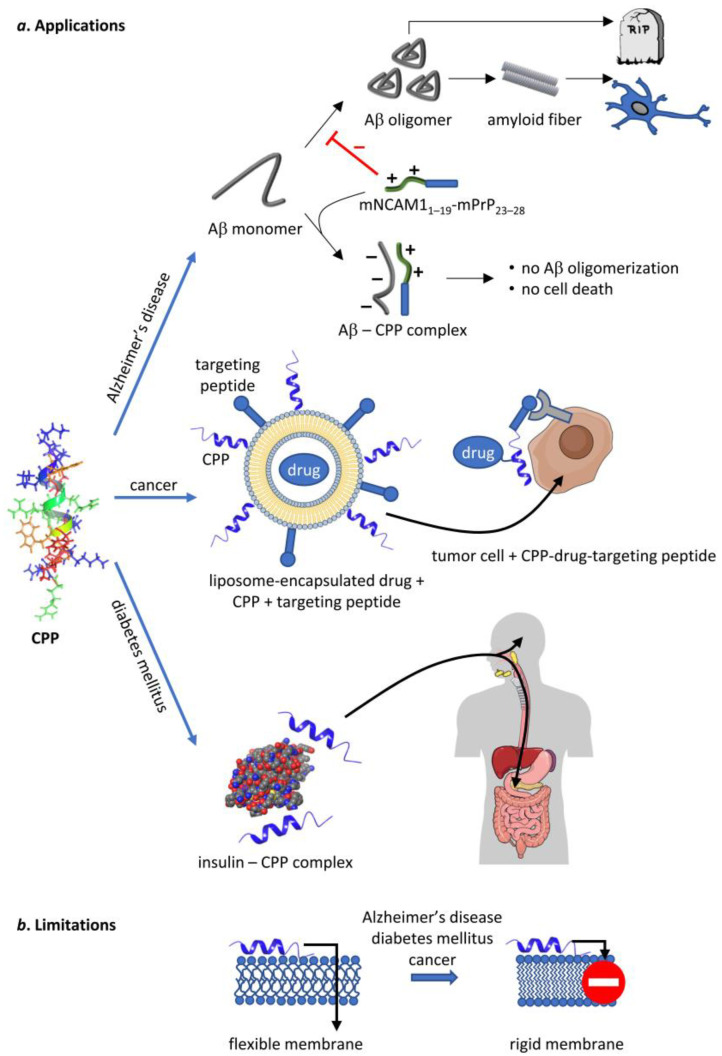
Potential therapeutic applications of CPPs and the limitations thereof. CPPs, represented by the NMR structure of penetratin in an artificial lipid bilayer [157], are capable of delivering therapeutic cargo or act on their own in several human diseases. They inhibit Aβ oligomerization in Alzheimer’s disease on their own, whereas they can be used to deliver chemotherapeutic drugs and insulin in cancer and diabetes mellitus, respectively. Unfortunately, all of these diseases are associated with such changes in membrane structure that inhibit the uptake of CPPs thereby limiting their therapeutic potential. Parts of the figure were created in the Mind The Graph platform (mindthegraph.com, accessed on 30 March 2020).

CPPs are undergoing intense preclinical and clinical evaluation in diseases like cancer, inflammatory diseases, amyotrophic lateral sclerosis, spinal muscular atrophy, neurodegenerative diseases, and ischemia [141,142,143]. Since the topic has been reviewed elsewhere [138,144], the aim of the current chapter is to collect the literature data showing that in diabetes, Alzheimer’s disease, and malignant diseases (i) alterations in the biophysical properties of membranes contribute to disease symptoms; and (ii) the clinical potential of CPPs is limited in these diseases by alterations in the lipid profile owing to the very disease the CPP is intended to cure (Figure 5).

### 7.1. Diabetes Mellitus

CPPs can enhance noninvasive transmucosal delivery of anti-diabetic drugs, which can be utilized in oral applications or intranasal delivery to the systemic circulation or even directly to the brain. Application of insulin complexed with different CPPs (Tat, oligoarginine, penetratin) resulted in their substantially increased absorption via transcytosis without causing detectable damage in cellular integrity [145,146,147]. When screening intestinal absorption enhancement of insulin by different penetratin analogues, the presence of positively charged Arg and Lys residues, chain length, hydrophobicity, basicity and amphipathicity of the CPP, and pre-complexation pH were all found to contribute to the efficiency [148,149].

Nasal administration is a particularly convenient method due to the large surface area of the highly vascularized nasal mucosa enabling fast and direct delivery of molecules. Besides uptake to the systemic circulation occurring in the respiratory region of the nasal cavity avoiding first-pass metabolism, delivery through olfactory and trigeminal routes bypassing the blood–brain barrier can directly transport drugs to the cerebrospinal fluid and the brain [150]. The first report laying the foundations of CPP application in intranasal insulin delivery to the systemic circulation showed that various CPPs can improve in vivo bioavailability of insulin in rats. L-penetratin was found more efficient than D-penetratin and octa-arginines without causing detectable damage to cellular integrity [151]. Subsequently, modified penetratins showed even better in vivo delivery efficacies that were comparable to that of subcutaneous administration [152,153].

An interesting aspect of CPP-facilitated intranasal delivery is the transport of anti-diabetic peptides directly into the brain. This can be particularly relevant in Alzheimer’s disease that is fundamentally associated with insulin resistance, impaired insulin receptor signaling, and dysfunctional glucose metabolism in the brain, which can contribute to the cognitive decline [154]. While insulin is mainly found in the systemic circulation after intravenous application, and nose-to-brain delivery of insulin alone has very low efficiency, intranasal coadministration of insulin and L- or D-penetratin resulted in significantly elevated insulin levels even in the distal regions of the brain in mice [155]. These effects of L-penetratin were associated with retarded progression of mild cognitive dysfunction in mice modeling amyloid overproduction [156].

One of the major limitations of nasal delivery is low efficiency of absorption [150], which can be significantly enhanced by conjugation of anti-diabetic drugs to CPPs. However, alterations in the composition and biophysical properties of membranes associated with diabetes, such as reduced deformability and elevated membrane rigidity [158,159,160], act against this advantage. The decreased membrane fluidity in diabetes is caused by elevated levels of cholesterol, ceramide and saturated fatty acids, and reduced amounts of unsaturated fatty acids [161,162,163]. The pathogenic role of these changes was emphasized by longitudinal studies demonstrating that higher saturated fatty acid levels and membrane rigidification precede the incidence of diabetes and can be considered as risk factors of the disease [164,165]. On the contrary, dietary substitution of saturated fat with membrane-fluidizing polyunsaturated fat is suggested to protect from diabetes [166,167]. These findings led to the formulation of the “membrane theory of diabetes”, according to which the amounts of saturated fatty acids increase in the cell membrane due to excess dietary intake or through conversion of consumed carbohydrates into saturated fats via lipogenesis. This route leads to compromised membrane fluidity and lipotoxicity resulting in the damage of pancreatic beta cells, attenuated insulin secretion and signaling, which contribute to the development of type 2 diabetes [160].

While the exact pathophysiological details are still not explored, adiponectin receptors (ADIPORs) were recently demonstrated to be involved in the intrinsic connection between diabetes and rigidification of the cell membrane. It has long been known that low levels of adiponectin and reduced ADIPOR signaling may contribute to damage of pancreatic beta cells, insulin resistance, and eventually, diabetes [168]. ADIPOR1 and ADIPOR2 proteins recently emerged as evolutionarily conserved regulators of membrane homeostasis by acting as membrane fluidity sensors to regulate phospholipid composition through promotion of fatty acid desaturation and incorporation of polyunsaturated fatty acids into membrane phospholipids. Recent studies in mammalian cells found that knockdown of ADIPOR2 (and to a lesser extent ADIPOR1) resulted in excess lipid saturation and membrane rigidification [169,170,171]. The proposed ceramidase activity of ADIPORs can additionally contribute to their membrane-fluidizing actions by lowering membrane ceramide and elevating sphingosine-6-phosphate levels [168,172]. Compromised membrane fluidity can in turn aggravate impaired insulin resistance by inhibiting secretion of insulin- or GLUT-containing secretory granules [173,174,175], impaired insulin receptor signaling [176,177,178,179,180] and increased membrane packing in the ER inducing ER stress leading to further saturated lipid synthesis [181,182,183].

Besides the involvement of membrane lipid alterations in precipitating diabetes symptoms, the higher rigidity of membranes of diabetic patients is also expected to reduce CPP-mediated penetration of anti-diabetic drugs by both the energy-dependent and -independent uptake routes (according to principles outlines in Section 4 and Section 6). Since this supposition has not been directly tested, future preclinical studies should address this correlation.

### 7.2. Alzheimer’s Disease

CPPs are promising therapeutic candidates in human amyloid-related diseases caused by the accumulation of peptides and proteins that form neurotoxic β-sheet-rich amyloid fibrils in their aggregated state [184,185]. Such potential effects were initially proposed based on the observation that mPrP_1–28_, the N-terminal peptide of mouse prion protein, shows strong similarity to CPPs, and can indeed act as a CPP and carry a large conjugated hydrophilic avidin protein into a variety of cells [186]. In prion diseases such as scrapie, Creutzfeldt–Jakob disease, or kuru, the normal α-helical isoform of prion protein PrP^C^ is converted into the aggregation-prone and pathogenic β-sheet-rich PrP^Sc^ isoform [187]. The CPP-like mPrP_1–28_ consisting of a hydrophobic signal peptide (mPrP_1–22_) and a highly cationic hexapeptide sequence (mPrP_23–28_, KKRPKP) was shown to effectively interfere with the PrP^C^-PrP^Sc^ conversion and prion propagation in cell cultures [188], which was lost in the absence of the N-terminal signal residues [188,189]. These effects were proposed to occur through non-receptor-mediated transmembrane cellular entry of CPP-like constructs supposedly leading to their binding to PrP^Sc^ via the polycationic motif, presumably in the endosome recycling compartment, which hinders further conversion of PrP^C^ into the misfolded form [184,189].

Alzheimer’s disease, the most common neurodegenerative disorder, is pathogenically linked to the accumulation of association-prone amyloid-β (Aβ) peptides originating from the amyloidogenic cleavage of the β-amyloid precursor protein (APP), a non-amyloidogenic, physiological cellular protein, through sequential proteolysis mediated by β- and γ-secretases residing in lipid rafts of the plasma membrane and in endolysosomal compartments. Aβ accumulation leads to the formation of smaller, strongly neurotoxic intermediate oligomeric and larger insoluble fibrillary aggregates, which enhance further aggregation in a prion-like autocatalytic cycle as described by the amyloid cascade hypothesis [190,191,192]. Consistent with the similarities between prion proteins and Aβ peptides, the mNCAM1_1–19_-mPrP_23–28_ CPP-like construct was shown to stabilize Aβ in an amorphous, non-amyloid aggregated state through direct interactions, and to effectively reduce Aβ oligomer concentrations, fibrillation and Aβ-induced toxicity in cellular studies. Another mNCAM1_1-19_-based construct carrying a different cationic hexapeptide (KKLVFF), derived from the KLVFF Aβ_16–20_ recognition sequence, exerted similar effects [193]. Subsequent studies confirmed that mNCAM1_1–19_-mPrP_23–28_ inhibited amyloid formation from Aβ peptides due to formation of specific heterooligomeric complexes between the positively charged hexapeptide of the construct and the negatively charged Aβ [194].

Plasma membrane composition and integrity are closely interrelated with the formation of amyloid oligomers and their neurotoxicity. Aβ oligopeptide aggregates seem to partially mediate neuronal cell death through compromising membrane integrity in Alzheimer’s disease. In keeping with the structural similarities with CPPs or AMPs, Aβ can interact with biological bilayers by similar mechanisms as described in Section 3. According to the widely accepted amyloid-pore hypothesis, during their aggregation process, Aβ oligopeptides can form annular protofibrils, amyloid pore-like structures exhibiting channel-like activity similar to that of AMPs leading to consequent permeabilization and elevated calcium levels contributing to cell death [195,196,197,198,199,200]. Several studies reported that apart from acting as channels, amyloid oligomers can also bind to the membrane surface and induce thinning, lipid packing disruption, and concomitant non-specific increases in the general conductivity of model bilayers in the absence of channel-like pores [201,202,203]. In addition, a detergent-like process was also raised to explain the cytotoxicity of Aβ aggregates. According to the suggested two-step model, pore generation can occur through Aβ oligopeptides inducing membrane curvature and forming metastable toroidal pore structures that, when collapsing, can lead to sequential disintegration of the membrane into peptide–lipid aggregates similarly to the mechanism induced by AMPs. Alternatively, amyloid fibrils growing on the membrane surface can pinch lipids and incorporate them into the developing fibril resulting in large-scale nonspecific fragmentation of the membrane [199,201,204].

Since amyloidogenic APP processing is initiated by lipid raft-dependent processes, raft disruption and reduced proximity of components of the amyloidogenic pathway induced by decreased cholesterol levels result in lower Aβ levels and neurotoxicity both in vitro and in vivo, which provides the basis of their potential therapeutic application in Alzheimer’s disease, as recently reviewed [205,206,207].

However, membrane lipids affect not only the production of Aβ but also its conversion into toxic aggregates. A preferential binding of the peptides to certain membrane lipids can induce molecular crowding leading to increases in the local peptide concentration favoring aggregation [201]. In general, Aβ weakly interacts with pure phospholipid bilayers, however, the presence of anionic phospholipids, such as PS or PG, facilitates membrane binding of Aβ [208,209]. The thickness and spontaneous curvature of model phospholipid bilayers was also shown to modulate aggregation propensity and conformation [210,211]. Cholesterol, besides affecting Aβ through lipid raft mediated modulation of enzyme activities, can enhance Aβ membrane binding, aggregation, and pore formation; however, these effects depend on bilayer composition and organization [212,213,214,215]. Gangliosides, raft-resident glycosphingolipids, were shown to cooperate with cholesterol and efficiently modulate Aβ aggregation. For example, GM1 ganglioside provides a more hydrophobic surface and therefore acts as a “seed” for aggregation by binding Aβ through its aromatic moieties and increasing its β-sheet content [216,217,218,219].

The pathophysiological relevance of alterations in membrane lipid levels in Alzheimer’s disease is further emphasized by clinical studies demonstrating positive correlation between hypercholesterolemia and the incidence of the disorder [220,221] and the beneficial effects of statin therapy [222,223]. Beyond changes in cholesterol level, reduced amounts of polyunsaturated fatty acids, PC, PE, and PS lipids, and elevations in saturated fatty acids, sphingomyelin, and ceramide species were described in Alzheimer’s disease [224,225]. Lipid alterations may also aggravate symptoms by inducing oxidative stress, demyelination, and altered neuronal signaling [224]. In addition, based on the intrinsic connection between membrane biophysics and CPP permeation, these changes can be expected to hinder the delivery of therapeutic CPPs to the affected neurons and their anti-amyloid effectivity. This assumption is worth testing in the future, so that more efficient CPP-based anti-Alzheimer therapeutics could be designed, which could add a novel approach to the scarce field of potential therapies.

### 7.3. Tumors

CPPs have the ability to carry cargos to target cells with high specificity and efficiency at low concentrations, and in some cases with selective cellular targeting. In contrast, the applicability of conventional chemotherapeutics is often limited by inadequate tissue or cell type selectivity leading to significant toxicity to healthy cells and low permeability through mucosal or cellular barriers resulting in poor efficiency. A comprehensive overview of studies describing the applicability of CPPs in tumors is beyond the scope of the current paper; therefore, we refer to excellent recent reviews in the field [226,227,228,229,230,231,232] and limit our discussion to the basic features of CPP-mediated antitumor approaches, which will be followed by a brief analysis of general alterations in the membrane lipid compositions and biophysical parameters of tumor cells, which may reduce the efficiency of CPPs.

A wealth of in vitro and in vivo experimental studies demonstrated the applicability of CPPs in models of malignant diseases [226,227,228,229,230,231,232] leading to testing these compounds in clinical trials to diagnose or treat cancer. In diagnostics, conjugation of p28, a tumor-targeting CPP, with a nontoxic indocyanine green fluorophore can provide a clear intraoperative visualization of tumors [233,234]. CPPs were also tested in delivering contrast agents for medical imaging [235,236,237,238,239]. Subsequently, AVB-620, an intravenously administered, fluorescent peptide dye conjugate containing a matrix metalloprotease-activatable CPP, effectively identified breast cancer metastases to lymph nodes in a phase I clinical trial (NCT02391194) [240].

Various CPPs entered preclinical and clinical trials in malignant diseases to test their therapeutic efficacy. p28, a fragment of Pseudomonas azurin protein, was shown to arrest tumor growth by inhibiting the ubiquitination and proteasomal degradation of p53 in vitro and in phase I clinical trials (NCT00914914 and NCT01975116) [241,242,243,244]. Reactivation of mutated or malfunctioning p53 was reported to be induced by a peptide derived from the C-terminal regulatory domain of p53 fused to Tat resulting in increased lifespan in an in vivo model of terminal peritoneal carcinomatosis and peritoneal lymphoma [245].

A major hurdle of antitumor agents is the unwanted toxicity in healthy surrounding tissues that can be improved by combining CPPs with homing peptides or ligands. For example, fusion of an anti-HER-2/neu peptide mimetic to Tat and a signal transducers and activators of transcription 3 (STAT3)-inhibiting peptide successfully decreased nonspecific uptake and increased specific uptake into ErbB2/HER2 positive cancer cells, which was accompanied by inhibited tumor growth in mouse xenografts [246]. As integrin α_V_β_3_ is commonly abundant in the plasma membrane of malignant cells, its ligand, the RGD (Arg-Gly-Asp) sequence, can drive selective tumor targeting as demonstrated by facilitated blood–brain barrier penetration and uptake of paclitaxel-loaded liposomes coated with an octa-arginine-RGD multifunctional peptide [247]. The tumor specificity of CPP–cargo complexes can also be increased by the natural targeting ability of antibodies against tumor-specific or tumor-associated antigens. For example, such a strategy was successfully applied in various in vivo studies in mice demonstrating selective targeting of colorectal cancer using a complex of Tat conjugated to an anti-carcinoembryonic antigen (CEA) monoclonal antibody [248]. An alternative strategy to improve tumor specificity of CPPs is to design activatable CPPs that are shielded, for example, by negatively charged counterparts or PEG chains until reaching a certain environment associated with the tumor, and then, get exposed. Such a condition can be the acidic pH typical for tumors or the activity of matrix metalloprotease (MMP) enzymes, especially MMP-2/9 that were demonstrated to induce CPP activation in several experimental systems [249,250].

Further increases in therapeutic efficiency of CPPs could be provided by manipulating the membrane lipid composition of neoplastic cells, which still represents an unexploited approach. Changes in the lipid composition of cellular membranes are common features in malignant diseases. Indeed, such alterations are often intrinsically involved in cancerous transformation. While the particular changes associated with the malignant transformation are variegated, some common trends emerge due to the generally elevated lipid uptake and reactivation of de novo fatty acid and cholesterol biosynthesis. This reprogramming leading to complex metabolic dysregulation is typically mirrored in abnormal activity of key enzymes of lipid metabolism, such as fatty acid synthase, acetyl-CoA carboxylase, ATP-citrate lyase, and transcription factors like Sterol Regulatory Element Binding Proteins (SREBPs), Liver-X Receptors (LXRs), and Peroxisome Proliferator Activated Receptor (PPARs). These changes tend to occur in the early stages of neoplastic transformation and substantially promote tumor progression in oxygen- and nutrient-depleted environmental conditions [251,252,253,254].

Changes of practically all major lipid classes were already described in various tumors. Among phospholipids, levels of PC species are typically elevated in cancer cells, which is advantageous due to metabolic flexibility as a result of the presence of a highly energetic material [255,256]. PS, normally localized into the cytoplasmic leaflet of the plasma membrane, is often exposed to the exofacial leaflet and is more abundant in neoplastic compared to normal cells, which induces a tolerogenic response in surrounding immune cells [257,258]. On the contrary, malignant cells are typically characterized by decreased PE content, which modifies spontaneous curvature of bilayers [259,260]. Furthermore, an increased ratio of saturated vs. unsaturated acyl chains in phospholipids and sphingolipids is commonly observed due to shifts form lipid uptake to de novo fatty acid synthesis [261,262].

The connection between cholesterol and tumors is underlined by the generally observed increased cancer risk in hypercholesterolemia, and the beneficial effects of statins in neoplastic disorders. Furthermore, cholesterol levels are increased in tumors due to a complex dysregulation of proteins involved in its uptake, efflux, and biosynthesis. The higher amount of cholesterol leads to enhanced proliferative, migratory, and survival signaling, among others due to excessive prenylation. Cholesterol conversion into oxysterols facilitates the formation of the immunosuppressed tumor microenvironment and escape from immune surveillance [254,263,264,265].

The aforementioned alterations contribute to changes in the biophysical properties of membranes. Modified physical properties of bilayers can in turn largely affect the structure and function of membrane proteins involved in tumorigenesis [10,254]. Increases in the levels of cholesterol and the fraction of lipids with saturated hydrocarbon chains significantly lower fluidity of cellular membranes, which is a general property of malignant cells, contributing to protection from both endogenous and exogenous insults. For example, increased bilayer rigidity can lead to multidrug resistance by inhibiting the uptake of chemotherapeutic drugs, by sequestration of drugs and by the activation of multidrug resistance-related ABC transporters. In addition, the accompanying reductions in the amounts of polyunsaturated acyl chains more susceptible to peroxidation protects tumor cells from oxidative damage and oxidative stress-induced cell death [253,263,266]. While the aforementioned changes in lipid levels observed in tumors are expected to increase the magnitude of membrane DP, which might induce activation of receptor tyrosine kinases, such as ErbB receptors [10,47,48], this assumption is not proven yet and has to be tested in the future.

It is reasonable to assume that a combination of the CPP-mediated delivery of antitumor agents and the application of therapies targeting the abnormal lipid homeostasis and modifying membrane lipid composition and structure can synergistically boost therapeutic efficiency of CPPs in cancer as well. Such therapeutic approaches, termed membrane lipid therapy, are recently emerging as effective alternatives in the treatment of cancer. They involve regulation of the biosynthesis and metabolism of membrane lipids; modification of bulk biophysical parameters and lateral nanodomain organization of the cell membrane; and approaches targeting tumor-associated molecules mainly through immunotherapy, as reviewed recently [126,254,264,267]. Given the roles of membrane biophysics in the modulation of CPP uptake, an appropriate selection of an optimized combination of these modalities is expected to provide a boost for anti-tumor CPP effects, which needs to be experimentally confirmed in the future.

## 8. Conclusions

Ever since their discovery, CPPs have been hailed as potential marvel drugs or drug carriers promising specificity and efficacy. Despite the well-founded nature of these expectations and the large amount of effort invested in their development, CPPs could not yet fulfill their promises. Besides the limitations rooted in the structure of CPPs leading to low bioavailability, short half-life, lack of specificity, and limited endolysosomal escape, we argue in this review that their efficiency is also strongly influenced by the membrane lipid environment that alters the biophysical properties of the plasma membrane. The lipid landscape of membranes are modified in several diseases leading to a more rigid membrane structure that decreases the membrane penetrating potential of CPPs.

The previous statement not only provides a further explanation for the failure or low efficiency of CPPs in clinical studies, but also suggests ways to overcome these limitations. Increasing the fraction of polyunsaturated lipids and decreasing the amount of membrane-rigidifying lipids, such as cholesterol and ceramide, increase membrane fluidity and are thereby expected to enhance the cellular uptake of CPPs. Furthermore, correction of the metabolic abnormalities leading to altered lipid composition of membranes must also be considered. Although the correlations between membrane biophysical properties and CPP uptake have already been firmly established in the past decades, the effect of systematic modifications of the membrane structure on the potential of CPPs to deliver therapeutic cargo into living cells in vitro and in vivo is waiting to be explored.
